# Effectiveness of two educational interventions based on the theory of planned behavior on oral cancer self-examination in adults: a randomized controlled trial

**DOI:** 10.1186/s12903-024-04315-5

**Published:** 2024-05-17

**Authors:** Anoosheh Ghasemian, Katayoun Sargeran, Mohammad Reza Khami, Ahmad Reza Shamshiri

**Affiliations:** 1 Research Centre for Caries Prevention, Dentistry Research Institute, Tehran University of Medical Sciences, Tehran, Iran; 2 Department of Community Oral Health, School of Dentistry, Tehran University of Medical Sciences, Tehran, Iran

**Keywords:** Mouth cancer, Self-examination, Primary prevention, Early detection of cancer, Educational models, Theory of planned behavior

## Abstract

**Background:**

The theory of planned behavior (TPB) is recognized as an effective theory for behavior change. The aim of the present study was to investigate the impact of two TPB-based educational interventions on oral self-examination (OSE) behavior and the related TPB constructs among adults in Tehran, Iran, in 2022.

**Methods:**

This randomized controlled trial involved 400 healthy individuals recruited from 20 urban comprehensive health centers in the southern part of Tehran, Iran. The health centers were randomly assigned to two control (PowerPoint) and intervention (WhatsApp) groups (200 individuals in each group). In the control group (the recipient of the routine care), participants received a 20-minute lecture through a PowerPoint presentation and a pamphlet. In the intervention group (the recipient of an additional intervention alongside the routine care), participants were educated through messages and images on WhatsApp along with having monthly group discussions. Data was collected at baseline, as well as at 1- and 3-month follow-ups using a structured questionnaire. The outcomes assessed included OSE behavior and the related TPB constructs: intention, attitude, subjective norm, and perceived behavioral control. Linear and logistic generalized estimating equations (GEE) regression models were used to evaluate the impact of the interventions with STATA version 17.

**Results:**

Of the total participants, 151 (37.75%) were men. The mean ± standard deviation (SD) of ages in the PowerPoint and WhatsApp groups were 39.89 ± 13.72 and 39.45 ± 13.90, respectively. OSE and the related TPB constructs showed significant differences between the groups at the 1-month post-intervention assessment. The effect of PowerPoint was more significant in the short-term (one month), while both methods showed similar effectiveness after three months, specifically in relation to OSE and the TPB constructs. At the 3-month post-intervention assessment, there were significant increases in OSE (OR = 28.63), intention (β = 1.47), attitude (β = 0.66), subjective norm (β = 2.82), and perceived behavioral control (β = 1.19) in both groups (*p* < 0.001).

**Conclusions:**

The present study provides evidence of the effectiveness of both educational interventions in improving OSE and the TPB constructs after three months. Therefore, both TPB-based educational methods can be recommended for designing and implementing interventions aimed at preventing oral cancer.

**Trial registration:**

The trial protocol was registered in the Iranian Registry of Clinical Trials (IRCT) on 04/03/2022 (registration number: IRCT20220221054086N1).

## Background

Cancers of the lip, oral cavity, and oropharynx collectively constitute oral cancer [1], a deadly disease when not diagnosed early and left untreated [2]. Globally, in 2020, there were 377,713 new cases and 177,757 deaths attributed to lip and oral cavity cancers [1]. According to the GLOBOCAN 2020, Iran had an estimated 1,139 new cases and 454 deaths related to lip and oral cavity cancers [3]. The increased morbidity and mortality associated with oral cancer can be attributed to a lack of knowledge and delay in diagnosis [4]. The stage of diagnosis for oral cancer has been identified as the most critical factor influencing survival rates and prognosis [5]. Given that most oral tumors are detected at an advanced stage, improving survival rates and prognosis hinges on primary prevention and early detection [4, 6].

Efforts to prevent and control oral cancer have been emphasized in the Crete Declaration on Oral Cancer Prevention [7] and by the World Health Organization (WHO), which advocates for strengthened health education, preventive behaviors, and early diagnosis of oral cancer [8]. Oral cancer can be primarily prevented by avoiding tobacco and alcohol consumption, promoting socioeconomic status (SES), increasing fruit and vegetable consumption, and encouraging oral self-examination (OSE) [9]. OSE has been recognized as an effective tool for the early detection of oral cancer if performed accurately by individuals [10]. Additionally, despite the average age for oral cancer occurrence is around 60 years [2], there has been a recent increase in its prevalence among young adults [11]. Hence, it is essential to educate individuals across various age groups about preventive behaviors. In this context, the success of oral cancer prevention can be significantly enhanced by implementing health education programs, which are well-established and practical methods for informing the public about the disease and motivating them to change unhealthy behaviors [12].

The effectiveness of educational programs is greatly influenced by the use of appropriate behavioral theories and models. These theories provide a foundation upon which educational interventions can be designed to bring about sustainable behavior change [13]. The theory of planned behavior (TPB) is primarily focuses on structures associated with individual motivational factors, which determine the probability of performing a behavior. The basic assumption of TPB is that the best predictor and the most important determinant of a behavior is intention. Behavioral intention indicates the intensity of willingness and effort to perform a particular behavior. Intention is predicted by three conceptually independent constructs. Attitude toward behavior reflects the favorable or unfavorable assessment of performing a certain behavior by an individual. Subjective norm shows that performing or not performing a particular behavior is influenced by social pressure from important people whether they agree or disagree with the behavior. Perceived behavioral control refers to the capability and control of an individual to perform a specific behavior, considering the degree of difficulty and existence of obstacles [13, 14].

TPB has been widely applied in numerous studies evaluating educational interventions targeting various health behaviors [15]. There are a few alternative behavior change theories and models that mostly utilized to investigate health behaviors, such as the health belief model (HBM), transtheoretical model (TTM), and social cognitive theory (SCT) [13]. TPB is a parsimonious and very useful model to explain and predict behavior [13, 16], and known as an effective theory based on which educational interventions can lead to maintainable change in behavior [13]. This theory is recognized for its effectiveness, offering concise and popular constructs that can be accurately measured using a well-defined guideline [17].

One of the main strengths of TPB is that it provides an excellent framework for identification of influential factors on behaviors with definite causal associations between the model components. Demographic and environmental factors are also considered via the constructs of theory with no independent contribution to explain the probability of performing a behavior [13]. Additionally, the TPB offers a dual-step method incorporating complementary qualitative to quantitative approach of individual believes which produces evidence-based results for educational interventions. It cannot only be used as a theoretical foundation of evaluating interventional programs, but also be utilized in conjunction with other educational theories and models to design behavior change interventions [13]. However, the limitation of TPB is that it does not account for the gap between intention and behavior in some situations that there is an improved intention with no persistent performance of behavior [18]. This problem can be addressed through the use of planning to change the behavior [19].

A number of epidemiological studies have evaluated the effect of educational interventions based on the TPB on oral cancer-related behaviors for prevention of oral cancer. However, all of the previous studies have been focused on examining tobacco smoking (cigarette, hookah), as a main oral cancer-related risk factor [20–25]. Moreover, some other studies have assessed the impact of TPB-based interventions on oral health-related behaviors [26–31]. On the other hand, OSE, as an important oral cancer-related preventive behavior, should be strongly promoted among the general public, especially within high-risk populations [32]. According to the Mouth Cancer Foundation, individuals aged 16 years and above should perform OSE monthly [33]. This behavior is critically important not only for the early diagnosis and treatment of oral cancer [34] but also for reducing its incidence and mortality rates [35]. To the best of our knowledge, no study has yet examined the impact of a TPB-based educational intervention on OSE. Only a few epidemiological studies have assessed the influence of educational interventions on OSE, either based on the HBM [35, 36] or without employing any behavioral models [37, 38]. Theory-based interventions are more effective in changing behavior compared to non-theory-based ones [39].

Considering insufficient knowledge about the importance of performing regular OSE for early detection of oral cancer, and lack of education on how to perform an accurate OSE based on the scientific instructions, there is an urgent need to conduct an educational program. Using an effective health education and promotion model, such as TPB, to evaluate OSE, especially among vulnerable individuals from low socioeconomic backgrounds who are at higher risk of oral cancer [40] is essential. We applied TPB, a behavior change model, that provides a theoretical basis for assessing behavior change interventions to design the study and develop the interventions, content, and measurement tool in order to answer the research questions.

The primary and secondary research questions were whether the educational interventions make significant changes in behavior and the related TPB constructs, respectively, during the study period [13, 14]. The findings underscore the importance of a TPB-based comprehensive educational program for prevention of oral cancer. The aim of the present study was to assess the effect of two educational interventions based on the TPB in improving OSE and the related TPB constructs among an adult population aged 15 years and above in Tehran, Iran, in 2022.

## Methods

### Study design and sample

**Fig. 1:**
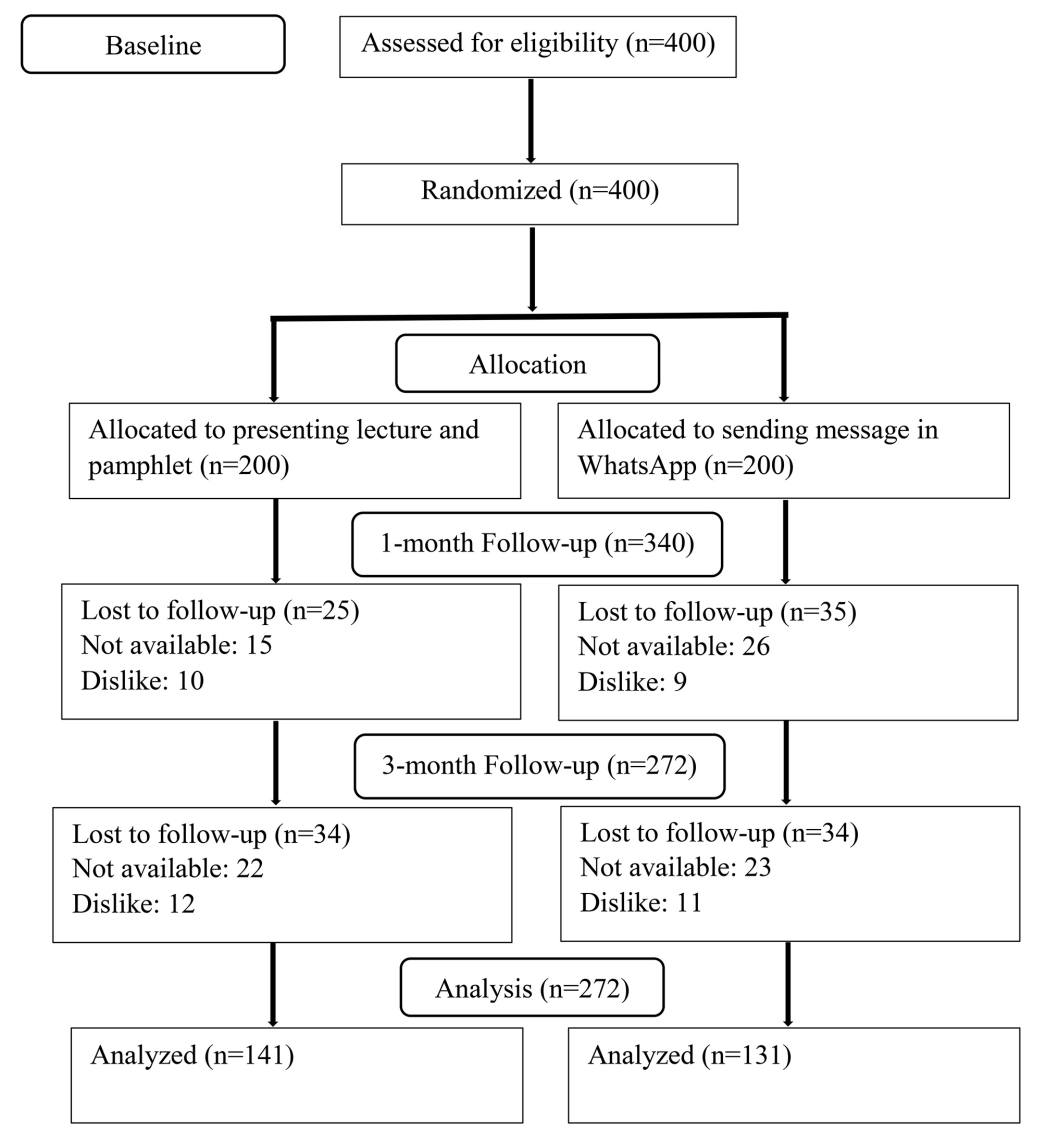
Flow diagram of the trial

The present study was a cluster randomized controlled trial (RCT) with parallel groups (allocation ratio 1:1) conducted in urban comprehensive health centers in the south of Tehran, Iran, from August 11 to November 25, 2022. The flowchart of the study is depicted in Fig. 1, with a total sample of 400 individuals from the general population under the coverage of the health centers initially enrolled. At the 1-month follow-up, 41 individuals were unavailable and 19 ones were unwilling to participate in the study, leading to 340 individuals to be analyzed. At the 3-month follow-up, 45 individuals were unavailable and 23 ones were unwilling to participate in the study, resulting in 272 individuals to be analyzed.

Participants in the study were aged 15 years and above, residents of Tehran in low-privileged or very low-privileged regions, healthy individuals with no underlying medical conditions, and literate, with at least basic reading and writing skills. They were also required to have a smartphone with the WhatsApp application installed, and their participation was contingent upon signing an informed consent form. Non-Iranian citizens, those who declined to participate in educational sessions or use social networks, and individuals unable to operate smartphone applications were excluded from the study.

### Sample size

Using the RCT formula with an equivalence design and considering parameters such as a type I error of 0.05 (z 1-alpha/2 = 1.96), power of 0.9 (z 1-beta = 1.28), delta of 1 (representing the difference and clinically acceptable limit), standard deviation (SD) of 2 (representing the combined SD of both comparison groups) [25], design effect of 2, and dropout rate of 20%, the estimated sample size was 400 individuals.

$$N = 2 \times {\left( {\frac{{{z_{1 - \frac{\alpha }{2}}} + {z_{1 - \beta }}}}{{{\delta _0}}}} \right)^2} \times {s^2}$$

### Randomization and blinding

The participants were selected through a simple cluster random sampling method. Given the study’s focus on a population with low SES, low-privileged and very low-privileged regions of Tehran were chosen from five regions dividing based on the socioeconomic development index [41]. Out of the 85 comprehensive health centers in these two regions, 20 centers were randomly selected. Simple randomization was conducted by generating random numbers using Microsoft Office Excel software to allocate 10 centers to the control (PowerPoint) group and 10 to the intervention (WhatsApp) group. To ensure allocation concealment, unique codes were assigned to the centers and groups, which were recorded in separate columns. Subsequently, a researcher who was blinded to the allocation performed the randomization based on these columns. Given the total sample size of 400, 20 individuals were randomly selected from each health center using the list of covered individuals. These selected individuals were then contacted by health workers and invited to participate in the study. The data analyst remained blind to the health centers and groups, with unique codes used for entry into the STATA software.

### Educational interventions and content

The both groups received the interventions, and the PowerPoint group served as the active control of the WhatsApp group. The control (PowerPoint) group received the routine care of health centers, while the intervention (WhatsApp) group received an additional intervention alongside the routine care. In the control (PowerPoint) group, participants received a 20-minute PowerPoint presentation comprising 20 slides with educational messages and colorful images in one lecture session held on a specific day and time to ensure that everyone attends. They also received a supplementary pamphlet containing a brief version of the same messages and colorful images of the PowerPoint slides. In the intervention (WhatsApp) group, participants received information via smartphones through 20 JPG images containing the same messages and colorful images as the PowerPoint slides converted into the JPG format to be able to send with WhatsApp. These images (each containing three to four messages and images on average) were sent to the participants on a specific day and time to ensure everyone had the opportunity to read them. It should be mentioned that the WhatsApp messages and images were delivered to the participants once a month along with having monthly group discussions. All participants were encouraged to review the pamphlet and WhatsApp messages regularly at home (twice a week) over the three months.

The messages and images of both the PowerPoint and WhatsApp groups were pretested in a pilot study conducted within a two-week period in two comprehensive health centers not included in the main sampling, on 40 individuals aged 15 years and older who were randomly selected (20 individuals in each center). Intervention for the PowerPoint group was performed in one center, and intervention for the WhatsApp group in the other. The messages and images were provided to the individuals to evaluate any problems or difficulties, as well as practical methods to solve them, or to amend any parts according to the results of the pilot study.

In both groups, the educational interventions included group discussion and question-and-answer at the end of the session, and once per month, respectively. Each participant received a dental package containing a toothbrush and toothpaste as an incentive to encourage their continued participation in the study. Additionally, in both groups, the interventions involved reinforcement reminder phone calls conducted at regular intervals (every two weeks) to maintain communication with participants, supervise their use of the educational materials, and provide an opportunity for participants to ask questions or seek clarification.

The total program in each group took 40 min. The lecture session in the PowerPoint group took 40 min, including 20 min for PowerPoint presentation, 10 min for distribution of pamphlet and explanation about the importance of reading it on a regular basis, and 10 min for group discussion and question-and-answer. In the WhatsApp group, the estimated time for creation of the WhatsApp group, sending the educational messages and images, and group discussion and question-and-answer was 40 min.

The participants were accurately educated on how to perform an oral cancer self-examination, which involved following eight steps [33] (Table 1).

**Table 1:** Eight steps of oral cancer self-examination [33]

**1- Look at your face**: Examine your face to see if there are any swellings you have not noticed before. Inspect your skin to see if anything has changed recently. Turn your head from side to side. This stretches the skin over the facial muscles to see lumps or bumps more easily.
**2- Check your neck**: Run the balls of your fingers under your lower jaw and down the large muscles on either side of your neck. Feel if there are any swellings, lumps, or bumps. Take note if everything feels the same on both sides.
**3- Examine your lips**: Pull your upper lip upwards and bottom lip downwards using your index, middle fingers, and thumb to look and feel inside of your mouth for any sores or changes in color. Use your thumb and forefinger to look and feel around and inside your lips for any lumps, bumps, or changes in texture.
**4- Feel around your gums**: Place your thumb and forefinger on the inside and outside of the gums of either the top or bottom teeth and work your way around the gums to feel for any lumps, bumps, or unusual textures.
**5- Peek inside your cheeks**: Open your mouth and pull your cheek to the side with your forefinger. Look inside for any red or white patches. Use your forefinger or tongue in the cheek to feel around for any ulcers, lumps, bumps, tenderness, sore areas, or rough patches. Repeat on the other cheek.
**6- Take a look at your tongue**: Pull out your tongue and look at each side for any ulcers, sores, swellings, or changes in color. Lift the tip of your tongue to the roof of your mouth to examine the underside of your tongue.
**7- Check the floor of your mouth**: Lift your tongue and look underneath at the floor of your mouth for any changes in color or sores. Gently press your finger along the floor of your mouth and the underside of your tongue to feel for any lumps, bumps, swellings, ulcers, or painful areas.
**8- Examine the roof of your mouth**: Open your mouth and tilt back your head to check the roof of your mouth. Look for any changes in color, sores, or ulcers. Use your finger to feel for any changes in texture.

The educational content of all materials (PowerPoint, pamphlet, and WhatsApp) was developed in collaboration with professors specializing in community oral health and health education and promotion, drawing on the existing literature and the TPB constructs. To do this, we searched the scientific literature emphasizing on studies based on the TPB [20–31] and scope of the study regarding OSE [4, 10, 35–38] to explore the detailed information required to be included in the content for each theory construct. Furthermore, we considered professional knowledge and scientific information of the specialists to prepare each part of the content in details.

The outline of educational content was firstly designed by the expert panel comprised of professors specializing in community oral health and health education and promotion. They determined the detailed information required to be provided in each part of the content for each theory construct, and explained all the details to the principal investigator in order to assure that the prepared content would be valid and effective. Next, the first draft of educational content was written by the principal investigator. To ensure more on its validity and effectiveness, it was reviewed and revised several times to achieve agreement of the specialists.

The content focused on the structures of the TPB and included strategies and messages aimed at reinforcing behavior and behavioral intention. It also aimed to promote attitude toward behavior, subjective norm, and perceived behavioral control related to regular OSE. The educational content emphasized the role of individuals in improving their perspectives and attitudes toward the behavior, as well as enhancing their ability and control over performing the behavior. Additionally, it highlighted the role of family and friends in fostering approval and expectations regarding the behavior.

Briefly, attitude incorporated advantages of performing regular OSE and disadvantages of not performing regular OSE, as well as messages about believing that it is beneficial and good to perform regular OSE. Subjective norm incorporated the effects and roles of society, culture, family, and friends in confirmation and support of performing regular OSE, as well as messages to family and friends for confirmation and support of performing regular OSE. Perceived behavioral control incorporated methods to increase the ability to perform regular OSE, as well as messages about being easy and having the ability to perform regular OSE.

### Training of health workers

Twenty volunteer health workers, one from each center, were invited to attend a training session where they were educated by a dentist through a lecture presentation using PowerPoint slides. The session covered information about the educational interventions, content, and guidelines for delivering educational materials to the participants.

### Data collection

#### Measurement tool and outcomes

No valid and reliable questionnaire based on the TPB was available in the field of oral cancer. Therefore, a two-section interviewer-administered questionnaire was structured based on the scientific literature and the concepts of the standardized TPB questionnaire [42]. The participants were interviewed by the principal investigator to complete the questionnaire. The first part included questions related to sociodemographic variables, such as age, sex, educational level, occupation, household income, marital status, housing status, household size, and family history of cancer. The second part included questions related to behavior, the primary outcome, and the TPB constructs, the secondary outcomes, with direct measurements.

The questionnaire was designed in two stages. At the first stage (design), it had 25 questions; nine questions about sociodemographic variables, two questions about behavior, and 14 questions about the TPB constructs. At the second stage (face and content validity), one question about subjective norm with content validity ratio (CVR) less than 0.75 was deleted, and the other question was edited. Additionally, two questions about perceived behavioral control were edited and merged into one question due to their similarity. Thus, the final questionnaire had 23 questions.

The outcomes were considered according to the following items:

### **Behavior**

Two questions were designed to assess OSE and its frequency:

- “Do you regularly inspect your mouth for possible oral lesions?” (Possible answers: Yes, or No).
- “How often do you inspect your mouth?” (Possible responses: daily, weekly, or monthly).

Subjects who inspected their mouth at least once per day, week, or month were defined as regular oral self-examiners.

For measuring the TPB constructs, three statements were created for each of them:

### Behavioral intention

- “I intend to inspect my mouth regularly for possible oral lesions within the next six months.“
- “I try to inspect my mouth regularly for possible oral lesions.“
- “I plan to inspect my mouth regularly for possible oral lesions within the next month.”

### Attitude toward behavior

- “Inspecting my mouth regularly for possible oral lesions is beneficial and valuable to me.“
- “Inspecting my mouth regularly for possible oral lesions is pleasant to me.“
- “Inspecting my mouth regularly for possible oral lesions is good to me.”

### Subjective norm

- “Most people who are important to me and whose opinions I value (e.g., family, friends, dentist, and doctor) confirm that I should inspect my mouth regularly for possible oral lesions.“
- “It is expected of me (by family, friends, and society) that I inspect my mouth regularly for possible oral lesions.“
- “I feel under social pressure to inspect my mouth regularly for possible oral lesions.”

### Perceived behavioral control

- “It is possible for me and if I wanted I could inspect my mouth regularly for possible oral lesions.“
- “I have complete control over inspecting my mouth regularly for possible oral lesions.“
- “It is mostly up to me whether or not I inspect my mouth regularly for possible oral lesions.”

A five-point Likert scale ranging from 1 to 5 was used to score all the statements, where “1 = I completely disagree,” “2 = I disagree,” “3 = I have no idea,” “4 = I agree,” and “5 = I completely agree.” Each construct was scored within a range of 3 to 15.

The structured questionnaire underwent pretesting before the study to assess its validity and reliability. Eight professors specializing in community oral health, health education and promotion, and oral diseases evaluated the face and content validity. One question with CVR less than 0.75 was deleted [43]. As all the questions had content validity index (CVI) higher than 0.79, no question was eliminated [44]. In a pilot study conducted in two comprehensive health centers not considered part of the main sampling, 40 individuals aged 15 years and older were randomly selected and invited to assess the reliability within a two-week period. The Cronbach’s alpha coefficients for behavioral intention, attitude toward behavior, subjective norm, and perceived behavioral control were 0.73, 0.80, 0.82, and 0.75, respectively. The intra-class correlation coefficients (ICC) for these measures were 0.99, 1, 0.98, and 0.99, respectively.

### Ethics

The protocol and all study procedures were approved by the Research Ethics Committee of Tehran University of Medical Sciences (ethical code: IR.TUMS.DENTISTRY.REC.1400.189). The research was conducted in accordance with the principles and guidelines of the Declaration of Helsinki. Written informed consent was obtained from all participants before their involvement in the study. For individuals aged 15 to 18 years, written informed consent was obtained from their parents and/or legal guardians. Subjects were assured that they could withdraw from the study at any time and that their personal information would be kept confidential.

### Data analysis

Categorical variables were presented as numbers and percentages (%), while continuous variables were described using mean and SD, as they were found to have a normal distribution through graphical and statistical methods. Chi-square test was used to compare participants who did or did not dropout from the study at the 1-and 3-month follow-ups regarding the baseline characteristics (attrition analyses). Multiple linear and logistic generalized estimating equations (GEE) regression models, utilizing an exchangeable correlation structure, were employed to assess the intervention effects on continuous and categorical outcomes, respectively. The models included group, time, and group-by-time interaction adjusting for all sociodemographic variables to remove residual confounding. In the GEE approach, every observation of each participant was considered in the analyses regardless of possibility of dropout that the participant had for later times. As we had no evidence that missing data was not missing completely at random (MCAR), the missing data was considered MCAR which did not introduce bias. The GEE approach is robust to missing data when data are MCAR [45], the fact that no systematic differences exist between participants with missing data and those with complete data [46]. Therefore, we did not utilize data imputation approach and eliminated unobserved data which did not impact our findings. Elimination of non-observed data seems more conservative than reproducing the same pattern that already exists in data using imputation methods. A p-Value < 0.05 was considered statistically significant. All statistical analyses were conducted using STATA version 17 (StataCorp, College Station, TX, USA).

## Results

Baseline characteristics of the participants revealed that the mean ± SD of age was 39.67 ± 13.80 years. Out of the total individuals, 249 (62.25%) were women. The majority of participants in both groups had achieved an educational level equivalent to a high school/diploma. Most of the individuals were housewives and married, owned their own houses, and did not have a family history of cancer. In the control (PowerPoint) group, the majority of participants lived in households of four people, whereas in the intervention (WhatsApp) group, households typically consisted of one to three people (*p* = 0.74). In the PowerPoint group, most participants fell into the medium income bracket, while in the WhatsApp group, most participants were in the low income category (*p* < 0.001) (Table 2).

**Table 2:** Baseline characteristics by groups among a sample of adults living in deprived areas of Tehran, Iran

	Group		
	Control (PowerPoint)	Intervention (WhatsApp)	p-Value
Age	39.89 ± 13.72	39.45 ± 13.90	0.75
Sex			
Male	80 (40%)	71 (35.50%)	0.35
Female	120 (60%)	129 (64.50%)	
Educational level			
Middle school and less	49 (24.50%)	44 (22%)	0.76
High school/Diploma	91 (45.50%)	98 (49%)	
Associate Degree and more	60 (30%)	58 (29%)	
Occupation			
Employee/Labor	30 (15%)	31 (15.50%)	0.91
Freelance/Self-employed	42 (21%)	35 (17.50%)	
Student	17 (8.50%)	20 (10%)	
Housewife	98 (49%)	102 (51%)	
Retired/Unemployed	13 (6.50%)	12 (6%)	
Household income			
Very low	38 (19%)	25 (12.50%)	**< 0.001**
Low	42 (21%)	75 (37.50%)	
Medium	66 (33%)	38 (19%)	
High	54 (27%)	62 (31%)	
Marital status			
Single/Widow	38 (19%)	37 (18.50%)	0.90
Married	162 (81%)	163 (81.50%)	
Housing status			
Personal	116 (58%)	113 (56.50%)	0.76
Rental	84 (42%)	87 (43.50%)	
Household size			
1–3	81 (40.50%)	88 (44%)	0.74
4	85 (42.50%)	82 (41%)	
5–7	34 (17%)	30 (15%)	
Family history of cancer			
Yes	54 (27%)	59 (29.50%)	0.58
No	146 (73%)	141 (70.50%)	

The comparison of dropout and non-dropout participants at the both follow-ups on sociodemographic variables indicated that there were no significant differences between the groups and no identified pattern was found.

At baseline, among 86 (21.50%) participants who performed regular OSE, 20 (5%) performed it at least once per day, 37 (9.25%) per week, and 29 (7.25%) per month.

There was an improvement in the percentages of OSE and the mean scores of intention, attitude, subjective norm, and perceived behavioral control in both the control and intervention groups throughout the study (Table 3).

**Table 3:** Percentages of oral self-examination (OSE) and mean scores of the Theory of Planned Behavior (TPB) constructs at baseline, one-, and three-month follow-ups by groups among a sample of adults living in deprived areas of Tehran, Iran

	Group					
	Control (PowerPoint)			Intervention (WhatsApp)		
	Baseline	First follow-up	Second follow-up	Baseline	First follow-up	Second follow-up
OSE						
Perform	44 (22%)	104 (59.43%)	125 (88.65%)	42 (21%)	74 (44.85%)	115 (87.79%)
Not perform	156 (78%)	71 (40.57%)	16 (11.35%)	158 (79%)	91 (55.15%)	16 (12.21%)
Intention	3.38 ± 0.60	4.47 ± 0.63	4.89 ± 0.31	3.35 ± 0.57	4.26 ± 0.62	4.86 ± 0.33
Attitude	4.43 ± 0.43	4.94 ± 0.15	4.98 ± 0.11	4.33 ± 0.39	4.88 ± 0.22	4.97 ± 0.10
Subjective norm	2.03 ± 0.86	3.79 ± 1.29	4.75 ± 0.66	1.90 ± 0.57	3.38 ± 1.13	4.74 ± 0.65
Perceived behavioral control	3.69 ± 0.53	4.59 ± 0.49	4.92 ± 0.25	3.70 ± 0.44	4.47 ± 0.51	4.91 ± 0.24

Overall, the attitude score for participants in the PowerPoint group was significantly higher compared to those in the WhatsApp group (*p* = 0.002). There was a significant difference between the groups regarding OSE and all the TPB constructs at the 1-month follow-up; however, these differences were not significant at the 3-month follow-up, except for attitude, where a significant difference between the groups was found at the 3-month follow-up. The odds of performing OSE and scores of intention, attitude, subjective norm, and perceived behavioral control significantly increased among the participants in both groups at the first and second follow-ups compared to the baseline (*p* < 0.001) (Table 4).

**Table 4:** The effects of groups on oral self-examination (OSE) and the Theory of Planned Behavior (TPB) constructs over 3 months among a sample of adults living in deprived areas of Tehran, Iran

	OSE		Intention		Attitude		Subjective norm		Perceived behavioral control	
	OR (95% CI)	p-Value	β (95% CI)	p-Value	β (95% CI)	p-Value	β (95% CI)	p-Value	β (95% CI)	p-Value
Group PowerPoint (vs. Group WhatsApp)	1.03 (0.63,1.68)	0.91	0.03 (-0.09,0.15)	0.64	0.10 (0.04,0.17)	**0.002**	0.12 (-0.08,0.33)	0.23	0.09 (-0.09,0.10)	0.85
Time (vs. Baseline)										
First follow-up	3.11 (2.13,4.53)	**< 0.001**	0.88 (0.79,0.98)	**< 0.001**	0.55 (0.49,0.61)	**< 0.001**	1.46 (1.28,1.65)	**< 0.001**	0.76 (0.68,0.84)	**< 0.001**
Second follow-up	28.63 (17.04,48.12)	**< 0.001**	1.47 (1.37,1.57)	**< 0.001**	0.66 (0.60,0.72)	**< 0.001**	2.82 (2.62,3.02)	**< 0.001**	1.19 (1.10,1.27)	**< 0.001**
Group by time interaction (vs. Group WhatsApp by Baseline)										
Group PowerPoint by First follow-up	1.84 (1.09,3.13)	**0.02**	0.18 (0.05,0.31)	**0.007**	-0.04 (-0.12,0.04)	0.34	0.30 (0.04,0.56)	**0.03**	0.12 (0.01,0.23)	**0.03**
Group PowerPoint by Second follow-up	1.07 (0.52,2.20)	0.86	-0.04 (-0.18,0.10)	0.58	0.10 (0.07,0.18)	**0.03**	-0.13 (-0.41,0.15)	0.36	-0.01 (-0.13,0.11)	0.84

p-Values were derived from generalized estimating equations (GEE) adjusted for age, sex, educational level, occupation, household income, marital status, housing status, household size, and family history of cancer

**Fig. 2:**
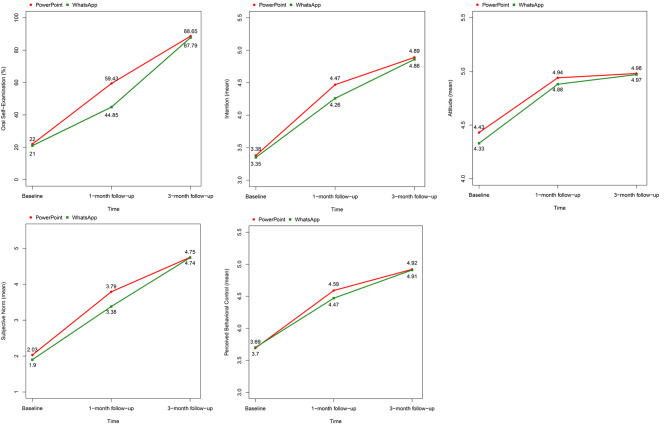
Trends of changes in oral self-examination (OSE) and the Theory of Planned Behavior (TPB) constructs by groups

As shown in Fig. 2, the PowerPoint group had a higher percentage of OSE and mean scores of the TPB constructs at the 1-month follow-up, but the percentages/means were quite similar between the groups at the baseline and 3-month follow-up.

## Discussion

This research was the first study aimed at evaluating the effectiveness of two TPB-based educational interventions in improving OSE and the TPB constructs after three months in adults aged ≥ 15 years in low-income areas of Tehran, Iran.

We found that OSE and the TPB constructs significantly differed between the groups. Although a difference was observed between the groups at the first follow-up, the difference was compensated at the second follow-up, indicating that both groups were similarly effective. Our results are consistent with two interventional studies that reported a significant impact of educational interventions on OSE [36, 37].

The results of the present study indicated that both educational interventions had significant influences on improving OSE over time, which is similar to the findings of previous studies conducted in Spain, Taiwan, Sri Lanka, and India [35–38]. Furthermore, both educational methods had significant effects on promoting intention, attitude, subjective norm, and perceived behavioral control after three months. A possible explanation could be attributed to the similar educational content delivered with the same messages and images, along with regular review of materials, cooperative group discussions, asking and answering questions, and reinforcement reminder phone calls, all of which were done with in-depth and precise supervision by the executive team during the study period. Additionally, the health workers, who had a similar socioeconomic and cultural background as the participants, were professionally trained to deliver the interventions, which could effectively contribute to a high level of motivation for promoting and committing to behavior change.

Improvements in behavior and all the TPB constructs over time can also be explained by the theoretical framework and specific strategies employed for each TPB component. The increase in OSE could be attributed to the TPB being an effective model for accurately assessing and sustainably changing behavior [13]. The explanation of key factors related to behavioral intention, such as intending, trying, and planning to engage in healthy behavior, along with motivation and encouragement to focus on the behavioral goal, led to an enhancement in intention. Participants were educated on the benefits of performing OSE, as well as the disadvantages of not doing so, resulting in an improvement in attitude toward the behavior. Furthermore, individuals were informed about the roles of society, culture, family, and friends as sources of social support, as well as the importance of social pressure to motivate OSE, leading to an increase in subjective norm. Additionally, participants were taught practical methods, such as creating a regular personal program to enhance self-efficacy and the ability to perform OSE and to develop self-control in performing OSE, resulting in an improvement in perceived behavioral control.

Our findings are consistent with some national and international TPB-based studies in which significant improvements were observed in various oral health-related behaviors and all constructs of the TPB after educational interventions [26–31]. In this regard, one study focused on different oral health-related behaviors, including tooth brushing, dental flossing, using fluoride mouthwash, and visiting a dentist regularly [26]. Two studies evaluated both tooth brushing and dental flossing [27, 29], and three others assessed only tooth brushing [28, 30, 31].

The Mouth Cancer Foundation has stated that, ideally, everyone should perform an OSE once a month [33]. Furthermore, anyone with any oral lesions persisting for more than two weeks in their mouth, after removing the causal factors, must be immediately referred to a healthcare professional for further consultation [47]. It has also been recommended that high-risk patients follow a 2-3-week rule for consultation with a healthcare provider [48]. The results of the present study demonstrated that most participants had never heard of inspecting their mouth regularly for possible oral lesions, and those who performed the behavior mostly did it on a weekly basis. The significant improvement in OSE after the interventions could be attributed to the fact that this behavior is feasible for anyone, as it is a simple, easy, and low-cost method for identifying and detecting oral cancer lesions at an early stage [10]. The enhancement of OSE in this study was greatly supported by the detailed training, including explanations and pictorial illustrations of the different steps, along with regular reminders, enabling the majority of participants to perform the behavior simply and regularly at home. The present findings might serve as a useful guide for dentists and oral health professionals to educate their patients on how to perform a thorough OSE and maintain it as an oral self-care habit, which could be an effective strategy for preventing and reducing the risk of oral cancer.

Several key strengths underpin the present study. First, the study was conducted with a substantial sample of men and women from the general population representing various age groups. Second, the educational interventions were conceptually based on a recognized and effective behavioral theory for assessing and changing behavior, which enhances the accuracy and reliability of the findings. Third, the use of both written and pictorial information for educating the participants, coupled with group discussions and repeated reminders, increased the attractiveness and effectiveness of the interventions. Fourth, the recruitment of trained health workers from the same background as the participants facilitated better communication and understanding. Fifth, the preventive interventions for OSE could be integrated into existing public health programs and professionally implemented by healthcare providers, effectively reducing the risk of oral cancer. Sixth, a structured questionnaire, considering the conceptual and methodological aspects of a standardized TPB-based questionnaire, was used to collect data, which was validated and pretested before the study.

The present study also had certain limitations. First, the study population was limited to urban low-income adults covered by health centers in the southern part of Tehran, potentially limiting the generalizability of the results. Second, the use of a self-reported questionnaire and the possibility of inaccurate answers due to personal and social concerns, may result in self-report bias. Lastly, as the evaluation period for the outcomes was only three months, future studies are recommended to extend the study duration to investigate the long-term effects of interventions on behavior change.

## Conclusions

The results of the present study indicated that both TPB-based educational methods were effective in improving OSE and promoting intention, attitude, subjective norm, and perceived behavioral control after three months. The effect of PowerPoint was more significant in the short-term (one month), while both methods showed similar effectiveness after three months, specifically in relation to OSE and the TPB constructs. These findings suggest that the TPB could serve as a comprehensive framework for designing and implementing educational programs focused on the prevention and early detection of oral cancer. This underscores the effectiveness of TPB as a behavior change model in the field of oral cancer prevention.

Future research could further leverage the TPB and its evidential results to design and conduct educational programs centered around OSE. It is also suggested that future studies use various training methods based on different behavioral models and extend the duration of study. It is advisable for oral health specialists and policymakers to prioritize the development of effective interventions and policies aimed at implementing preventive programs in the domain of oral cancer. Such efforts are essential for facilitating early detection, diagnosis, and treatment of this disease. The practical preventive strategies can be consequently integrated into the public health system and applied in systemic disease prevention programs using the common risk factor approach.

## Data Availability

The datasets used and analyzed during the current study are available from the corresponding author on reasonable request.

## Abbreviations

WHO: World Health Organization
SES: Socioeconomic status
OSE: Oral self-examination
TPB: Theory of planned behavior
HBM: Health belief model
TTM: Transtheoretical model
SCT: Social cognitive theory
RCT: Randomized controlled trial
SD: Standard deviation
CVR: Content validity ratio
CVI: Content validity index
ICC: Intra-class correlation coefficient
GEE: Generalized estimating equations
MCAR: Missing completely at random
